# Social support in the workplace for physicians in specialization training

**DOI:** 10.1080/10872981.2018.1435114

**Published:** 2018-02-21

**Authors:** Leena Mikkola, Elina Suutala, Heli Parviainen

**Affiliations:** ^a^ Department of Language and Communication Studies, University of Jyvaskyla, Jyvaskyla, Finland; ^b^ Faculty of Social Sciences/Health Sciences, University of Tampere, Tampere, Finland

**Keywords:** Continuing medical education, learning through service, social support, workplace interaction, workplace relationships, physicians in specialization training

## Abstract

When becoming a specialist, learning-through-service plays a significant role. The workplace affords good opportunities for learning, but the service-learning period may also impose stress on phycisians in specialization training. In medical work, social support has proved to be a very important factor in managing stress. Social support may afford advantages also for learning and professional identity building. However, little was known about how social support is perceived by doctors in specialization training. This study aimed to understand the perceptions of physicians in specialization training regarding social support communication in their workplace during their learning-through-service period. The study was conducted qualitatively by inductively analyzing the physicians’ descriptions of workplace communication. The dataset included 120 essays, 60 each from hospitals and primary healthcare centres. Physicians in specialization training explained the need of social support with the responsibilities and demands of their clinical work and the inability to control and manage their workloads. They perceived that social support works well for managing stress, but also for strengthening relational ties and one’s professional identity. A leader’s support was perceived as being effective, and both senior and junior colleagues were described as an important source of social support. Also co-workers, such as the individual nurse partner with whom one works, was mentioned as an important source of social support. The results of this study indicate that social support works at the relational and identity levels, which is due to the multi-functional nature of workplace communication. For example, consultation functions as situational problem-solving, but also the tone of social interaction is meaningful. Thus, strengthening one’s professional identity or collegial relationships requires further attention to workplace communication.

**Abbreviations** PiST: Physician in specialization training

## Introduction

Learning through service plays a significant role in becoming a medical specialist in Finland. The completion of a specialist degree requires five to six years of medical practice, further studies, including both medical and management content and, at minimum, nine months of service in public health centers.^1^ Learning through service occurs in medical practices, which are embedded in daily work routines, such as problem-solving, evaluation of diagnosis and treatment, understanding different opinions, and providing explanations [1]. The workplace affords good opportunities for learning, but creating a favorable learning environment requires attention. For example, an excessive workload threatens the learning of physician in training because of the severe stress it imposes on them [2].

A very important factor in managing stress has proved to be social support. Social support refers to social interaction in which resources are received from others [3]. Social support helps one manage uncertainty, increases one’s perception of personal control over one’s life experiences [4], and helps one toward goals [5]. In medical work, support from both leaders and co-workers is strongly connected to lowered job strain and improved health outcomes [6–9]. Social support can promote coping [10], diminish occupational stress [11,12] and reduce perceptions of depersonalization [13]. It can prevent psychiatric symptoms and common mental disorders [14,15] and has a protective function against possible suicidal intentions [16,17]. Low social support from co-workers is associated with emotional exhaustion [18,19]. Interestingly, when there are low co-worker support and limited possibilities to control one’s job, physicians are more likely to experience poor psychological well-being than other healthcare professionals [20]. Although social support is a major factor in physicians’ well-being and stress management, it can be a meaningful stress-buffering factor in workplace learning as well.

Social support can provide advantages other than stress management. Some studies indicate that social support significantly explains job satisfaction [21,22] and positively correlates with physicians’ organizational commitment and engagement [23]. There is also some evidence showing connections between social support and learning or professional development; there are correlations between social support and physicians’ work orientation [24], as well as general practitioners’ work practices [25]. There is some evidence that social support can develop working skills in patient–caregiver relationships [13], and social support can impact managerial learning [26]. Co-worker support is significant for professional efficacy [27] and self-efficacy [28]. Interestingly, social support in the workplace also impacts the choices junior physicians make between specializations [29,30]. Thus, social support seems to have a role in one’s professional development.

A physician’s professional identity is socially constructed through interaction in the workplace [31]. Professional identity can be seen as a representation of one’s self, which is formed over time in stages through an internalization of the norms, values, and characteristics of one’s medical profession [32]. Professional medical identity is dynamic, and it changes throughout one’s life and career [33]. When beginning one’s postgraduate medical studies, the process of positioning oneself as both a specialist and a future leader in the complex context of a healthcare organization becomes active [34]. This phase of medical studies calls for reflection, which is a central process in professional identity formation [35]. Thus, learning through service is an important arena for one’s professional identity formation, and role models and mentors have a crucial role in the process [36]. However, the medical workplace is a demanding organizational and interpersonal environment. Because workplace learning is in many ways mediated by workplace relationships with seniors, peers, other healthcare professionals, and patients, learning occurs through different discourses in the workplace [2]. Social support, which emerges from social interaction in the workplace, is also an important factor in identity building [37]. Thus, it is reasonable, and somewhat inevitable, to focus on social support and workplace communication during one’s learning-through-service period.

This study aimed to understand the perceptions of physicians in specialization training regarding social support communication in their workplaces during their learning-through-service period. Even though the importance of social support in medical work is well known, only a couple studies have examined the perceptions of physicians in specialization training to date. To achieve this study’s objective, three research questions were asked:

1. According to the perceptions of physicians in specialization training, what are the origins of needs for social support?
2. According to the perceptions of physicians in specialization training, what kinds of functions of social support are actualized in workplace communication?
3. According to the perceptions of physicians in specialization training, what kinds of relationships are supportive in the workplace?

These questions were answered qualitatively by analyzing physicians’ descriptions of social support in workplace communication during their learning-through-service period. In this study, social support was approached from a communication perspective [38]. Social support was defined as verbal and nonverbal communication that helps one manage uncertainty and increases one’s perception of personal control over one’s life experiences [4]. Thus, the focus was on workplace communication and physicians’ perceptions of them.

## Method

This study analyzed essays written by physicians in training during their specialization studies in Finland in the fall of 2012 and the spring of 2013. The essays were collected in a module on leadership interactions and organizational communication, which was part of multi-professional social and health management studies and leadership studies. The module was based on pre-reading materials, lectures, and an essay. The essay instructions were quite open. The participants were asked to describe their perceptions of communication practices and leadership communication in their workplaces by reflecting on the following question: what kinds of appropriate and inappropriate practices (i.e., patterns) of social interaction are there in your workplace? The physicians in specialization training were also asked to reflect on what is particular about leadership communication in healthcare organizations. No word target was set. The essays were submitted to the lecturer through an online learning environment. Altogether, 225 physicians in training finished the essay during their specialist training studies; 120 physicians in training were from hospital organizations and 105 physicians in training were from healthcare centers. The data included participants from 5 out of 22 healthcare districts in which healthcare is organized in Finland [39].

The dataset in the study included 120 essays (60 from hospitals and 60 from primary healthcare centers) that were three to seven pages long. The dataset was formed by selecting every other essay based on the essays’ order of arrival. The data were rich, and the dataset covered different kinds of workplaces in the fields of specialized care and primary healthcare. In Finland, hospital sizes vary from 300 to 10 000 employees working in the fields of different specialties. In primary healthcare centers, there are 100–2000 employees. The dataset included descriptions of both large and small organizations. Physicians with a wide variety of specializations, ranging from general medicine to psychiatry, were represented. On the basis of the essays’ content, one could posit that at least half of all possible (49) specialties were represented and 13 fields of specializing were directly mentioned in the dataset.

Ethical principles were followed carefully throughout the process [40]. The integrity of the research subjects was respected; extensive information about the study was given, participation was voluntary, and the participants gave written consent for their essays to be used for research purposes. No pressure was placed on the physicians in specialization training, and participation and non-participation did not have any consequences on the physicians’ studies. To protect the informants’ privacy, all personal information (e.g., names and localities of workplaces) was removed from every essay before the dataset was formed. The anonymity of the research subjects was attentively considered throughout the research process. Finland has a policy that states an ethical review is not usually needed when a study’s research subjects are adults and when the study does not expose them to exceptionally strong stimuli or possible harm [40]. Thus, no approval was sought for the study.

The data included both the writers’ reflections on their experiences and the writers’ perceptions of workplace communication. The essays discussed the topics of leadership communication and social interaction in the medical work. Social support was a recurring theme in the essays, so the study was focused on social support communication. Thus, the primary methodological perspectives and goal setting were inductive. The data were read many times to construct an overview. Then, all of the content related to social support was chosen. The initial selection was performed using key words such as support, social support, help, aid, advice, problem solving, ventilation, emotion, uncertainty, stress, and strain. Then, the essays were checked to ensure that all relevant content was selected.

An interpretive inductive analysis was conducted to build a qualitative description of the perceptions of physicians in specialization training [41,42]. The method was used to systematically describe the meanings included in this qualitative material [43] and to conduct a systematic process of identifying, coding, and categorizing the data [44]. The analysis proceeded by condensing and coding the data according to the process description presented by Graneheim and Lundman [45]. The analysis was conducted manually. First, the text was fractioned into meaning units, which were words or sentences that included one distinct meaning. The fractioning was focused on manifest content [45]. Next, the meaning units were condensed, and those condensed units were further abstracted into codes [45]. Thus, the data was abstracted, and a total of 111 codes were labeled. An example of this process of abstraction is presented in Table 1.

**Table 1.** Examples of the coding process

Original quote	Meaningful unit	Condensed meaning	Code	Category
‘I guess that in primary healthcare, the sense of lacking control over one’s own work and especially the workload is a big problem that people experience’.	‘I guess that in primary healthcare, the sense of lacking control over one’s own work and …’	The lack of control is problematic	Lack ofcontrol	The need for social support
	‘… especially the workload is a big problem that people experience’.	The heavy workload is problematic	Workload	

The categories were formed inductively but were guided by the research questions. The main categories were needs for social support, functions of social support and supportive relationships in the workplace. Each category included two to three sub-categories. In Table 2, the categories and frequencies of different types of comments are presented.

**Table 2.** The categories and comment frequencies

Categories	Contents	Sub-categories	Frequency
Needs for social support	Why is social support seen as important?	(1) Responsibilities and demands of clinical work	25
		(2) Inability to control and manage workloads	19
Functions of social support	What kinds of outcomes and forms does social support have?	(1) Stress-management functions	49
		(2) Relational functions	51
		(3) Identity functions	37
Supportive relationships in the workplace	What are the sources of social support?	(1) Leader–follower relationships	60
		(2) Collegial relationships	27
		(3) Co-worker relationships	23

The original data were read through to evaluate the coherence between the results of the analysis and the original essays and to confirm that the categories resembled the data. The reviewers’ backgrounds were in communication studies (one author) and health science (two authors). Thus, no researcher had a bias toward specialties, and each researcher observed the data from the position of an outsider of the medical workplace.

Finally, experiences described in the primary healthcare and hospital sectors were compared to identify differences between organizational types. However, the emphasis and results differed in only one theme: supportive relationships. Therefore, the results are presented together, and the difference is described in the results section.

The data were written and the analysis was conducted in Finnish. The results were written in English on the basis of Finnish categories and sub-categories; thus, some translating was done when this article was written. Quotes were chosen to describe the essence of each category during the writing process, and they were translated from Finnish to English at once. There was a challenge in conveying the spirit of each quote’s tone; however, the fact that the data was initially written in the form of essays made translating the quotes easier than translating spoken and recorded quotes [46].

## Results

### Perceived origins of stress in the workplace

Physicians in specialization training explained the need of social support with the (1) responsibilities and demands of their clinical work and (2) the inability to control and manage their workloads. They described the essence of the clinical work as a challenging task that demanded expertise and combined heavy responsibilities. The physicians illustrated this as challenging charges, dark feelings caused by patients’ suffering, severe cost of possible mistakes, burdensome decision making, demands for physicians’ work, and the loneliness of physicians’ work. Furthermore, they emphasized the heavy emotional load and distress of the work. A physician in specialization training (in examples the abbreviation PiST refers to physician in specialization training) described the demands of the work as follows:

In my opinion, especially in physicians’ work, social support is really important. Decision making and taking on responsibility are sometimes almost too heavy to carry on. The work is challenging and basic medical studies do not prepare a young physician to face this mental pressure. (PiST 31)

The physicians in specialization training recognized the gap between their earlier education and their shorter professional experience and the expectations they faced as physicians. The gap created a need for social support. Thus, uncertainty emerged from the mismatch between the physicians’ resources and challenging situations.

In addition, the inability to control and manage one’s own workload raised a need for social support. Thus, stressors originated from the organizational environment. Physicians in training described the stressors not only as ‘too much work’, but also as decisions made by someone else or at some other location, such as decisions about appointments. Such decisions took away the physician’s control over one’s job:

I guess that in primary healthcare, the sense of lacking control over one’s own work and especially the workload is a big problem that people experience. (PiST 28)

Physicians in specialization training saw that their work is strongly regulated by organizational structures; there is constant uncertainty in the environment and only a few possibilities to influence what they do and how they do it. Therefore, the needs for social support are focused on managing environmental stressors.

### Functions of social support in the workplace

Physicians in specialization training perceived that social support worked not only for managing stress, but also for strengthening relational ties and one’s professional identity. The functions were described in the physicians’ in training narratives of their positive and negative experiences during their learning-through-service periods and of the communication behaviors in which social support was enacted.

#### Stress-management function

The physicians in specialization training described social support as communication that reduces one’s stress, increases one’s sense of control, and enhances one’s mental well-being. The physicians in specialization training articulated social support, for example, as situational aid and specific shared information, such as practical tips; however, social support was also described more generally as a problem-solving tool. Discussing problematic cases, reflecting on knowledge, asking for and giving opinions, and sharing experiences were mentioned as the social support that helped the physicians in specialization training manage stressful situations and uncertainty.

More so than information, physicians in specialization training emphasized conversations and problem solving as being important. Even when aid and advice were mentioned, a two-way interaction process and reciprocity were emphasized. The basic form of this kind of support was consultation, which, at best, was described as solutions and decisions constructed in interactions. The following example describes the importance of consultation as social support:

Social support from my close superior is available. I think the deputy chief’s expertise is important, and I appreciate his competence. I appreciate the possibility of being able to consult [with him] on difficult cases. In the process of specialty training, it has been possible to get the deputy chief’s support every day. (PiST 6)

Social support’s stress-management function was described as problem-centered communication in which the focus of supportive interactions was on problem solving and information processing. However, physicians in specialization training also perceived that emotion-centered communication enhanced their stress management, even though they wrote relatively little about emotional support. They described emotional support as expressing emotions, communicating at an emotional level, and having an emotional and listening-based attitude. The following quote refers to the emotional content of communication:

Consultation in primary healthcare, at least when it occurs between two ‘equal’ colleagues, is often reflecting and sharing your own emotions and experiences. Thus, it works as supervision and it [sharing] is justified and especially needed in this mentally distressing work. (PiST 19)

#### Relational functions

Physicians in specialization training perceived that social support had a relational function. Social support was described as communication that strengthens workplace relationships by building team spirit, developing positive work environments, and even creating ‘passion for work’. It was also described as communication that maintains a ‘functional work community’ in which members can ‘get along’. The function was described as follows:

Feedback about your work and the social support provided by your superior helps you to prevent burnout, helps you to be flexible and engage with the organization and work better. (PiST 14)

The relational function was described to actualize through problem- and emotion-centered communication, but physicians in specialization training emphasized sharing as a form of social support that helps one reach relational outcomes. Sharing experiences and opinions, asking about moods and feelings, reaching a personal level in social interaction, expressing presence, showing interest, and showing appreciation and dignity were described as forms of communication that strengthened workplace relationships. According to the physicians in specialization training, those kinds of communication embodied togetherness and belonging:

We [colleagues working in same room] do things together and talk about things besides work-related issues. Our interaction is collegial [and] professional, but [also] warmer and more human than with our larger work community. Within our pack, you can laugh and cry [and] be good or bad. Our interaction is appropriate, and it enhances everyone’s mental well-being, but they also include practical tips for our daily work. (PiST 35)

The connection between a positive work environment and social support was seen as a two-way process. Social support created a positive environment that enhanced physicians’ in-training opportunities to receive more social support:

I feel that social support in our workplace is quite good. A good crew and team spirit as well as a willingness to help each other are the most important things when it comes to managing work stress. In our community, we have license to consult each other, and we feel this [consulting] is a resource and an important part of [our] interactions. (PiST 15)

##### Identity function

Physicians in specialization training described social support as empowering. They noted that the empowerment was actualized when appreciation was expressed. Such communication enhanced the sense of worth, honor and dignity. Empowering communication was described as communication that showed they were heard and cared for as persons. This is actualized in interpersonal communication, as illustrated by the next example:

She [the chief physician] generates trust and is interested in the well-being of her juniors [and their abilities to] manage work and work-related problems, which are often connected to patients in this job. For a young physician, it is very important how the chief physician reacts to requests for a consultation. (PiST 43)

The identity function was carried out by problem- and emotion-centered social support. However, more than just situational problem-solving and emotional distress-relief, functions were described as long-term effects of supportive communication. Physicians in specialization training referred to enhanced professional knowledge and professional learning, which improved self-directedness, as an important part of professional competence:

In gastro-surgery, the [social] support from supervisors has been remarkable … On Monday mornings, we start with an informal chat over coffee, in which all the patients operated on over the weekend and possible future operations are surveyed … [It’s] an important learning situation. The interaction is relaxed, and the physicians in training participate almost as equals. (PiST 19)

As the above example illustrates, social support is important for learning; furthermore, being ‘almost … equal’ is seen as social support. Such supports strengthen perceptions of professional ‘worth’ among specialists. Thus, the relational function is closely attached to identity function. Moreover, at best, such collegial discussions take place informally during the workday:

[Having an] easy, informal, reflective discussion with an experienced colleague and a supervisor is very important. The moments help many of us [physicians in training] go further. (PiST 35)

### Supportive relationships in the workplace

Physicians in specialization training perceived leader-follower relationships, collegial relationships, and co-worker relationships as potential sources of social support. They expressed expectations of social support from leaders and senior colleagues.

### Leader support

Leader support was characterized as how each leader was interested in his or her subordinates, handled the subordinates’ uncertainties, acted as a leader or mentor as needed, positioned him or herself as the subordinates’ defender, and acted as a resource for the subordinates. A supportive leader was described as someone who ‘stands for the league’. The following example is a description of a supportive leader:

A good leader also provides social support and communicates supportively; we [physicians in training] know how to do and manage, and we do important work. This signals that the junior’s contribution is valued and one’s well-being is cared for. (PiST 23)

The physicians in training perceived their leaders’ support as effective. The effectiveness was explained as leader’s ability to reduce situation-based uncertainty and to impact subordinates’ work. Physicians in specialization training expected social support from their leaders, and they described positive experiences when their expectations were fulfilled. However, also negative experiences, in which their leaders were distant and difficult to approach were described.

#### Collegial relationships

Each collegial relationship was based on a shared professional code and was seen as a potential source of social support. Both senior and junior colleagues were described as support sources. Social support from senior colleagues actualized in medical consultations. Experiences of the potentially supportive situations were twofold. Support was considered crucial and effective for one’s professional development, as in the following example:

There is an arrangement for young physicians in training … in which there is a possibility to consult [with] a specialist … [the] atmosphere is usually light and glad. We [specialists and PiSTs] introduce perceived problems and search for solutions together. (PiST 50)

However, the master–journeyman relationship (referring to the relationship between a skilled worker and a novice) was considered unsupportive if the advice was ‘patronizing’ and there was ‘no discussion’. The effectiveness of collegial support was based on professional knowledge, but physicians in specialization training noted that the effectiveness depended on how content was expressed and whether reciprocity was recognized in the senior–junior interaction.

Social support in collegial relationships with peers was described as solely positive. The supportiveness was based on similar medical knowledge and similar levels of competence, and physicians in specialization training found it easy to approach their peers. The following quote illustrates the meaning of collegial relationships with peers:

Daily short meetings with physicians help you manage to get on. You get updated on how everyone is doing, and [you] chat, for example, about challenging patients and difficult cases … [You and your peers] get support and advice. (PiST 6)

#### Co-worker relationships

For co-worker relationships, physicians in specialization training referred to either multi-professional relationships in their workplaces or teamwork. In particular, nurse partners were mentioned as important sources of social support. The support of co-workers was explained as appreciation at a personal level, which was perceived as strengthening one’s belonging to one’s clinical community. The following example describes the meaning of personal relationships:

… down the years, you often get to know someone you work closely with more privately … which makes it possible to share with them, to some extent, both private issues and issues that threaten well-being at work. (PiST 60)

Co-worker support was described positively, and co-worker relationships were most often mentioned by physicians in specialization training at primary healthcare centers, where collegial relationships can be rare.

## Discussion

This study aimed to understand the perceptions of physicians in specialization training regarding social support communication in their workplaces during their learning-through-service periods. The results showed that the physicians in specialization training perceived many functions of social support in their work. Considering earlier studies [11–13,18,19], it is not surprising that the stress-management function strongly emerged in the data. In several earlier studies, a covariation was revealed between physicians’ coping skills and social support [6–12]; this connection was recognized by the physicians in training as well. Stressors had origins in health organization and clinical work. Hence, one can conclude that physicians in training have both professional and environmental uncertainties in their work and workplaces. An earlier study [20] indicated that a lack of control of one’s work is associated with poor psychological well-being especially among physicians. Combined with perceived heavy responsibilities, a lack of control is straining. Because of their roles, physicians in specialization training have few opportunities to control the work environment while facing many expectations as clinicians and learners, and even role conflicts may emerge. Hence, this study’s findings suggest that the learning-through-service period is very stressful, but social support helps to manage the stress. This result emphasizes the importance of availability of social support in medical workplaces.

The results of this study also indicate that social support works at the relational and identity levels. Drawing a cautious conclusion, this observation is in line with earlier studies [27,28] in which a connection between social support and professional or self-efficacy was found. In this study, physicians in training described the functional – or rather multi-functional – nature of social support; when solving problems the relational and identity functions were also actualized. Early theoretical literature [47,48] showed that social support builds self-esteem and a perception of acceptance [49]. Nevertheless, especially in a work-life context, interest has been on merely stress buffering. In the context of medical workplace learning, however, both identity and relational functions are important, because they work toward the objectives of specialization training, toward professional identity formation [32]. Every senior is an important role model in the identity formation process [36], and it is extremely important to be aware that every professional encounter may reinforce or hinder the process of professional identity formation. Every supportive encounter also enhances resources to manage stress and that way it enhances learning.

The results of this study show that physicians in specialization training see both hierarchical and peer relationships as potentially supportive. They attribute the impact of leader support to a leader’s ability to influence straining work conditions. The support of collegial relationships is explained by professional similarities, which were noted as significant in earlier studies [50–52]. A new finding is the role of cross-professional co-worker relationships as a source of social support. In particular, in primary healthcare settings, nurse partners are considered important sources of social support, and the effectiveness of co-worker support is attributed to personal knowledge and sharing. Thus, even though cross-professional relationships may not support professional problem solving, they may play a role in job satisfaction and stress management. However, collegial relationships and medical leadership remain major sources of social support from the perspective of professional learning.

The findings of this study show that physicians in training do expect to receive social support from their leaders and senior colleagues. In earlier studies, supervisors and mentors were seen as important sources of social support [53,54]. However, this study’s results show that social support is not enacted in every workplace as expected. It is possible that the intention to provide support is not recognized in some workplace communications, but it is also possible that there are some contradictory or even unintentional messages that are interpreted as unsupportive. Earlier studies have showed that advice given as social support is more effective if the advice is communicated in a way that preserves dignity [55,56]. Furthermore, even if one’s intentions are good, there may be some unintended consequences. Earlier studies showed that methods and contexts of providing feedback influence learning [57,58]; hence, it is reasonable to suggest that situation and context of providing social support does that too. For example, in some situations providing support may threaten loss of face – a wish to be seen as competent or to be liked [59] – of a physician in training. Therefore, further attention in regards to workplace communication is needed. It requires time to build mutual trust in a workplace relationship, but it also requires knowledge and competence.

## Limitations

This was a qualitative research study. The content analysis was conducted systematically as described in the method section. The results were in line with those of earlier studies, so the dependability of the study was confirmed. Original quotes were presented to allow the reader to evaluate the credibility of the analysis [60]. The data itself were comprehensive and included rich descriptions of specializing physicians’ experiences of social support in their workplaces. The qualitative data provided the opportunity to understand how physicians perceive social support and supportive relationships in their workplaces. However, the participants in the study worked as physicians before their specialization training; therefore, the data may reflect some earlier experiences. Nonetheless, the experiences were all from the initial stages of the participants’ careers.

The essay instructions were broad in scope and did not focus solely on social support. Narrower instructions might have produced more detailed examples, and in further studies, it would be interesting to examine, for example, how support is sought in the workplace. Nevertheless, this study, especially with its inductive approach, showed the importance of social support in specialization training and the role of social support in workplace interaction. Future studies should focus on authentic communication practices in healthcare workplaces to develop an understanding of supportive communication behaviors.

## Conclusion

This study described the perceptions of physicians in specialization training regarding the social support they perceived during their learning-in-service periods. The physicians in specialization training saw social support as functional communication that helped them manage stress, which largely originated from their roles as ‘professionals and trainees’ and the gaps between their resources and expectations. They recognized that social support had relational and identity functions, which helped them achieve the goals of learning and form their identities. However, the availability of social support did not guarantee the enactment of social support.

Social support may work toward the objectives of specialization training, especially toward professional identity formation, and it is possible to integrate supportive communication with, for example, feedback. However, the quality of interpersonal relationships and interpersonal communication in the medical workplace needs further attention. Future studies should focus on actual communication in the workplace to gain a deeper understanding of what kinds of communication behaviors are effective when one is aiming to provide social support. Also, more focused research designs are needed to understand the relationship between supportive communication and professional identity.

## Note

1. In Finland, the basic medical education degree is Licentiate of Medicine, and it takes at least six and a half years to complete. When physicians graduate, they work under the supervision of a senior colleague. Full authorization to act as a general practitioner requires additional training in primary healthcare. To become a licensed medical specialist, one has to take part in continuing education; specializing physicians (i.e., physicians in training) complete further medical studies and five to six years of workplace learning as residents. In 2008, the Finnish curriculum concerning medical doctors’ specialization was updated, and management and leadership education was increased to 10–30 points of European Credit Transformation System (ECTS) [61].
